# Antisolvent precipitation for the synergistic preparation of ultrafine particles of nobiletin under ultrasonication-homogenization and evaluation of the inhibitory effects of α-glucosidase and porcine pancreatic lipase in *vitro*

**DOI:** 10.1016/j.ultsonch.2024.106865

**Published:** 2024-03-29

**Authors:** Xiaonan Zhang, Yan Huang, Siyi Huang, Wenyi Xie, Wenxuan Huang, Yi Chen, Qiyuan Li, Fajian Zeng, Xiongjun Liu

**Affiliations:** a Jiaying University, Meizhou 514015, China; b Conservation and Utilization Laboratory of Mountain Characteristic Resources in Guangdong Province, Meizhou 514015, China

**Keywords:** α-Glucosidase, Porcine pancreatic lipase, Ultrafine particles of nobiletin

## Abstract

To further enhance the application of nobiletin (an important active ingredient in *Citrus* fruits), we used ultrasonic homogenization-assisted antisolvent precipitation to create ultrafine particles of nobiletin (UPN). DMSO was used as the solvent, and deionized water was used as the antisolvent. When ultrasonication (670 W) and homogenization (16000 r/min) were synergistic, the solution concentration was 57 mg/mL, and the minimum particle size of UPN was 521.02 nm. The UPN samples outperformed the RN samples in terms of the inhibition of porcine pancreatic lipase, which was inhibited (by 500 mg/mL) by 68.41 % in the raw sample, 90.34 % in the ultrafine sample, and 83.59 % in the positive control, according to the data. Fourier transform infrared spectroscopy analysis revealed no chemical changes in the samples before or after preparation. However, the crystallinity of the processed ultrafine nobiletin particles decreased. Thus, this work offers significant relevance for applications in the realm of food chemistry and indirectly illustrates the expanded application potential of nobiletin.

## Highlights

• Ultrasonication-homogenization synergistically assisted antisolvent method was effective.
• Ultrafine nobiletin particle with a minimum particle size of 521.02 nm.
• Ultrafine nobiletin particle had better enzyme inhibition effect than raw.

## 1 Introduction

The poor solubility of functional compounds in food limits their ability to perform their intended functions, lowering their functional activity [1], [2]. To increase the activity of samples, these functional components can be processed and converted into ultrafine particles [3]. In addition to increasing a compound's specific surface area, a compound's particle size (<1000 nm) also improves its stability and biological performance [4]. Nobiletin is a natural polymethoxy flavonoid found in *Citrus* fruits [7], and it plays a crucial functional flavonoid role. A great deal of interest has been generated in the discipline of food chemistry through studies on its pharmacological activity, which mostly focus on the clinical auxiliary treatment of illnesses as well as its anti-inflammatory [8] and enzymatic inhibitory actions [9]. However, raw nobiletin (RN) is difficult for organisms to ingest because of its poor water solubility and low bioavailability [10].

To decrease the particle size of substances and increase bioavailability, a variety of technologies are being applied in the manufacturing industry, including loading polymers [5] and adding lipids, emulsions and chitosan [11]. The use of these technologies in food and other functional commodities is strongly constrained by the formulation complexes they employ [5]. Instead of compounds that are already loaded with certain chemical components, one often favors compounds with high solubility, permeability, and bioavailability in these situations. A comparably advantageous ultrafine technology is the antisolvent recrystallization technique [5], and studies have successfully produced ultrafine particles utilizing the antisolvent precipitation approach and confirmed the high solubility and biological activity of ultrafine particles. For example, Zhang et al. incorporated an additional ultrasonic device based on antisolvent precipitation and produced daidzein microparticles with high efficiency; an ultrafine powder with a particle size of 200–300 nm was obtained, and the free radical scavenging effect was also enhanced [12].

There are two types of ultrasound-assisted operations: direct and indirect (ultrasonic probe, combined water bath container-ultrasonic device) methods [13], [14]. The ultrasonic probe can be placed in direct contact with the material, avoiding energy consumption. Despite the reasonable degree of application success of the standard ultrasonic-assisted antisolvent precipitation method, there are still several drawbacks, including unevenly produced particles and a low rate of powder recovery. The separation of the water bath and the structural features of the container in the combined water bath-ultrasonic approach will also result in a reduction in the intensity of ultrasonic vibration applied to the material.

In this study, we synchronized the operation of a probe-type high-speed homogenizer and a probe-type ultrasonic device in an antisolvent environment, and ultrafine particles of nobiletin (UPN) were created by recrystallization in an antisolvent system by two driving forces. We attempted to utilize porcine pancreatic lipase [15] and perform an α-glucosidase inhibition experiment [16] to compare and confirm the activity of the samples. With these tests, we hoped to offer a solid foundation for the use of ultrafine technologies in real applications.

## 2 Materials and methods

### 2.1 Materials

Raw nobiletin (RN, purity > 98 %) was purchased from Shanghai Yuanye Biotechnology Co., Ltd. (Shanghai, China). Orlistat (purity > 97 %) and acarbose (purity ≥ 98 %) were purchased from Aladdin (Shanghai, China), and dimethyl sulfoxide (DMSO) and anhydrous ethanol were purchased from Tianjin Chemical Reagent Co., Ltd. (Tianjin, China, analytical grade) and from a Millipore water filtration system (Bedford, MA, USA).

### 2.2 Preparation of ultrafine particles

**Fig. 1:**
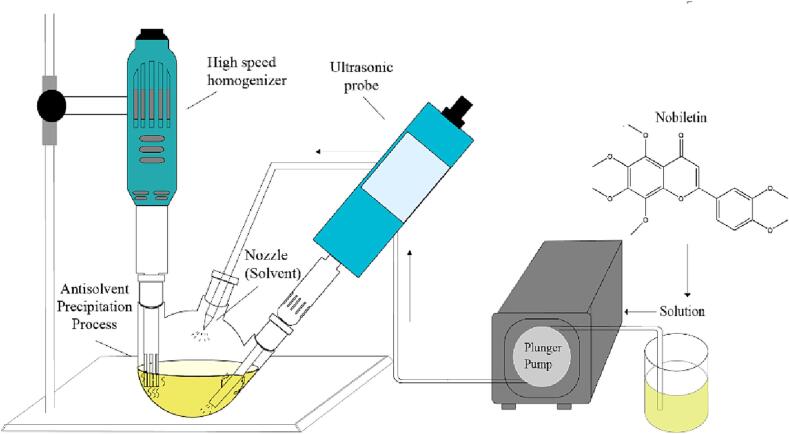
Ultrasound- and homogenate-assisted antisolvent process.

As shown in Fig. 1, in the process of preparing ultrafine particles, we used a KH 700DE ultrasonic probe (Wuxi, China; made of stainless steel; length × diameter: 125 × 7.5 mm; and the ultrasonic power range is 300–1000 W), a VS-25S homogenizer (Wuxi, China; the probe was made of stainless steel; length × diameter: 150 × 10 mm; and the homogenization speed range 5000–35000 r/min), and a GM-0.5B vacuum pump (experimental range 1–30 mL/min) was used in combination with the solvent system for nanoparticle preparation. In the experiment, a nobiletin solution (4–10 mg/mL) was transferred into a three-neck round-bottomed flask (capacity: 500 mL) with an aperture of 240 mm. The liquid flow rate was 6–12 mL/min. The solution was ultrasonicated at powers ranging from 0 to 300 W. The ratio of solvent to solution (liquid–liquid ratio) ranged from 5 to 25:1. The solvent temperature was maintained between 10 and 40 °C. The resulting paste was freeze-dried to obtain UPN, which was subsequently tested for its physical and chemical properties.

### 2.3 Optimizes the UPN particle size

**Fig. 2:**
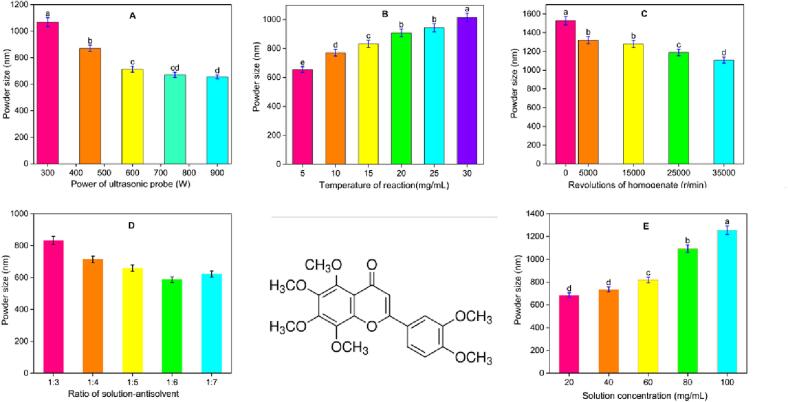
Influence of various factors on nobiletin particle size: (a) ratio of liquid to solution, (b) reaction temperature, (c) reaction of the homogenate, (d) solution concentration, and (d) processing power of the ultrasonic probe.

We investigated the influence of different factors on the particle size prepared by ultrasound- and homogenate-assisted antisolvent process. The single-factor experiment was repeated 3 times, and the average value was taken (As is shown in the Table 1 and Fig. 2). To refine the impact of the interaction between various variables on UPN size, we used the response surface BBD (using Design-Expert 13 software version) method for experimental optimization[17]. Based on the results of the single-factor experiment, we selected three factors that have a significant impact on particle size, namely, ultrasonic probe power (A), solution concentration (B) and homogenization speed (C), for the BBD experiments. Regression and analysis of variance were used to evaluate the impact of process parameters and plot the RSM.

**Table 1:** Analysis of particle size and standard deviation under different factors.

	**Ultrosonic** **Power (W)**	**Particle** **Size (nm)**	**Concentration** **(g/mL)**	**Particle** **Size (nm)**	**Ratio**	**Particle** **Size (nm)**	**Temperature** **(℃)**	**Particle** **Size (nm)**	**Revolation** **Homogenate** **（r/min）**	**Particle** **Size (nm)**
1	300	1068.2 ± 32.10a	20	685 ± 20.35d	1:03	833 ± 24.85a	5	654 ± 19.80e	0	1529 ± 45.40a
2	450	870.4 ± 23.42b	40	736 ± 21.86d	1:04	714 ± 21.75b	10	771 ± 23.05d	5000	1320 ± 39.30b
3	600	713.2 ± 21.75c	60	819 ± 24.75c	1:05	659 ± 19.95c	15	832 ± 24.95c	15,000	1280 ± 38.20b
4	750	671 ± 19.65 cd	80	1093 ± 32.20b	1:06	587 ± 17.80d	20	908 ± 27.10b	25,000	1189 ± 35.35c
5	900	655 ± 15.34d	100	1256 ± 38.05a	1:07	622 ± 18.30 cd	25	943 ± 28.10b	35,000	1107 ± 33.10d

### 2.4 Microscopic characterization of powders

Scanning electron microscopy (SEM) was used to analyze the morphology of the samples. Small amounts of RNs and UPNs were affixed to the double-sided copper conductive adhesive. A layer of nanometer metal powder was sprayed onto the prepared sample. After gold spraying, the homogenates were observed in a sample chamber with a current of 50 mA for 30 s or with a current of 3 mA for 6 min. The samples were compared with the RNs, and then, SEM images were captured under acceleration voltages of 15 kV and 20 kV.

### 2.5 FTIR analysis

In accordance with the methods of Zhang et al. [18], we accurately weighed 190 mg of KBr and 2 mg of sample powder, combined them completely, dried the resulting mixture for 2 min in an infrared drying oven, ground it in the same direction, and subsequently formed the powder into tablets. The results of an investigation using infrared scans with wavelengths between 400 and 5000 cm^−1^ were recorded.

### 2.6 XRD analysis

The crystal shapes of the nobiletin samples were examined using an X-ray diffraction (XRD) powder diffractometer (Philips, Netherlands). A 10 mg sample of nobiletin particles was evenly distributed on a glass slide for analysis. Cu K_α1_ radiation was applied at 30–40 kV. XRD patterns were recorded at 2θ. With a step size of 0.02° and a scanning velocity of 5°/min, the scanning range ranged from 10 to 70°.

### 2.7 Nuclear magnetic resonance (HMR) analysis

*An* AscendTM 400 (Bruker, China) nuclear magnetic resonance instrument was used to detect the raw powder and nanopowder of the samples. 1H NMR (400 MHz, DMSO‑*d*_6_) and 13C NMR (100 MHz, DMSO‑*d*_6_) spectra were obtained. ESI-MS was performed using an Esquire 3000 + mass spectrometer (Bruker Daltonics, Germany) equipped with a gas atomizer probe capable of analyzing ions up to *m*/*z* 6000. The mass spectrometer was operated in full scan mode, and it was positive.

### 2.8 Detection of nobiletin samples by LC-MS

After the generated powder sample was fully homogenized using ultrasonic treatment for 5 min, it was dispersed in deionized water. A 0.22 μm organic filter membrane was used for filtration, and an AB SCIEX API4000 type LC-MS/MS System was used for detection. The conditions of the experiment were as follows: The mobile phase was a solution of 0.1 % formic acid. The gradient elution method was used to inject 1 μL of methanol. The column temperature was 35 °C, and the flow rate was adjusted to 0.4 mL/min.

### 2.9 Activity detection of powders

#### 2.9.1 Experiment on the ɑ-glucosidase inhibition of nobiletin samples

The α-glucosidase inhibition experiment was performed according to the standard protocol, with some modifications [19]. RN and UPN were dispersed in deionized water and diluted to different concentrations (15.63, 31.25, 62.50, 125, 250, and 500 μg/mL). After complete ultrasonography, a 0.22 μm filter membrane was filtered for use. In 1 mL of phosphate buffer solution (0.1 M, pH 7.2), 2 μL of α-glucosidase was dissolved. The reaction substrate, 8 mmol/L 4-nitrophenyl-D-glucopyranoside (PNPG) solution, was made by adding 200 mg of powdered PNPG to 125 ml of phosphate buffer. First, 10.6 g of sodium carbonate was dissolved in 100 mL of deionized water. as a stopper. Different concentrations of the nobiletin sample solution (0.1 mL) and the α-glucosidase solution (0.1 mL) were mixed well (2 μL/mL), and each reaction mixture received 200 μL of PNPG solution. The reaction was terminated with Na_2_CO_3_. The sample absorbance was measured at 405 nm. The procedure's positive control was acarbose, which was used at the same concentration as the sample.

#### 2.9.2 Experiment on porcine pancreatic lipase inhibition of nobiletin samples

The RNs and UPNs were dissolved in deionized water at concentrations of 15.63, 31.25, 62.50, 125, 250, and 500 μg/mL, and a 0.22 μm filter was used after thorough ultrasonication. Six milligrams of porcine pancreatic lipase (PPL) was dissolved in 10 mL of buffered phosphate (0.1 M, pH 7.2). Then, as the reaction substrate, 8.4 μL of p-nitrophenylbutyrate (PNPB) was dissolved in 10 mL of acetonitrile. Different amounts of nobiletin sample solution (0.2 mL) were carefully mixed with 0.1 mL of PPL solution, and the mixture was then diluted to 2 mL with phosphate buffer. The samples were mixed evenly by ultrasonication and incubated at 37 °C for 30 min. An ultraviolet spectrophotometer was used to measure the absorbance at 410 nm, with orlistat serving as the positive control.

#### 2.9.3 Enzyme inhibition rate

$Enzyme\;inhibition\;\left(\%\right)\;=[1-\left(\frac{{\mathrm{Abs}}_{\mathrm{sample}}-{\mathrm{Abs}}_{\mathrm{sample}\;blank}}{{\mathrm{Abs}}_{\mathrm{control}}-{\mathrm{Abs}}_{\mathrm{control}\;blank}}\right)$] × 100 %.

where Abs _sample_ is the sample's absorbance value when it contains an enzyme and substrate solution; the absorbance of sample and substrate without enzymes is the Abs _sample blank_; and the absorbance of buffer is the Abs _control_. Substrate solution and enzyme; Abs _Control blank_ is the absorbance of the buffer and substrate without enzyme [20].

## 3 Results and discussion

### 3.1 Effect of each factor on the sample size

In this study, a higher liquid–liquid ratio can successfully decrease the size of UPNs, but a higher liquid–liquid ratio results in more solvent waste and more production-process losses. Fig. 2a illustrates how the liquid–liquid ratio affects the size of UPN particles. The smallest particle size of 587 nm was achieved when the ratio was 1:6; therefore, this ratio is appropriate for further research. According to some research, the slowing of molecular motion caused by a decrease in temperature prevents tiny molecules from colliding and sticking together in a solution system, resulting in the formation of small particles. The lowest particle size of UPN, 654 nm, was produced at 5 °C in this study, which was attributed to the effects of temperature conditions in the range of 5 to 30 °C (Fig. 2b).

When all the other variables were held constant, the particle size was negatively correlated with the speed of the homogenate in the reaction system. Fig. 2c shows that the particle size of UPN exhibited a decreasing trend in the 0–35,000 r/min reaction system. This may be because high-speed homogenization fully impacts and shears larger molecules in the two–phase solution system, leading to the production of more small molecules. High-speed homogenization is good for the synthesis of tiny molecules according to Yu et al. [12], which further supported the findings of this investigation. Fig. 2d shows the particle size of UPN obtained by changing the solution concentration while keeping the other conditions unchanged. As the concentration of solution increases, the particle size of UPN gradually increases, possibly due to the large concentration of solution leading to the production of more particles in the antisolvent process, which further increases the probability of collisions between particles and leads to adhesion and combination; eventually, the particle size increases [21]. In addition, too high a concentration will not only lead to an increase in production costs but also cause waste, so the selection range is optimized at a solution concentration of 20–60 mg/mL.

Particle size reduction is facilitated by an increase in the cavitation amplitude created per unit time and an increase in the sound intensity, both of which are directly correlated with the ultrasonic power [22]. The particle size of the sample has a negative relationship with the ultrasonic power when the power range is 300–900 W. At 300 W, the particle size is greatest (1068 nm), while at 900 W, it is lowest (655 nm). However, when the power reaches 750 W, the particle size gradually decreases (see Fig. 2e). The optimal ultrasonic power for further study is 750 W since a higher power results in higher energy consumption, which is incompatible with actual manufacturing.

### 3.2 Analysis of the response surface results

Using Design-Expert 10 software, multiple linear regression and quadratic polynomial fitting were carried out. Table 2 displays the variance analysis findings, with P < 0.01 indicating high statistical significance. The unknown components do not significantly affect the outcomes of the model fitting, and the model error is minimal. *R^2^* = 0.9596, indicating a good fit of the equation. The order in which each element had an impact on nobiletin particle size was X_1_ > X_3_ > X_2_. Particle size was significantly impacted by the major keyword X_1_ and the secondary terms X_1_^2^ and X_2_^2^ (P < 0.01).

**Table 2:** Box–Behnken design (BBD) with the experimental values for ultrafine particles of nobiletin (nm), analysis of variance (ANOVA) for the response surface quadratic model, and fit statistics for the response values.

**No**	**BBD Experiments**				**ANOVA**						
	***X_1_*** *(r/*min*)*	***X_2_*** *(mg/mL)*	***X_3_*** *(W)*	***Y_1_***	**Source**	**Sum of Squares**		**DF**	**Mean Square**	***F***	***p***
1	15,000	60	750	457.21	Model	3.538E^+05^		9	39309.71	18.45	0.0004*
2	15,000	40	900	688.68	*X****_1_***	66499.40		1	66499.40	31.21	0.0008*
3	25,000	60	900	539.38	*X****_2_***	3044.34		1	3044.34	1.43	0.2708
4	25,000	40	750	805.89	*X****_3_***	5406.96		1	5406.96	2.54	0.1552
5	25,000	20	900	750.65	*X****_1_****X****_2_***	521.44		1	521.44	0.25	0.6359
6	15,000	20	750	507.45	*X****_1_****X****_3_***	83593.27		1	83593.27	39.24	0.0004*
7	15,000	40	600	495.59	*X****_2_****X****_3_***	25806.82		1	25806.82	12.11	0.0103
8	35,000	60	750	688.32	*X****_1_^2^***	58698.19		1	58698.19	27.55	0.0012*
9	35,000	20	750	692.89	*X****_2_^2^***	81845.41		1	81845.41	38.42	0.0004*
10	35,000	40	900	555.97	*X****_3_^2^***	12991.10		1	12991.10	6.10	0.0429
11	25,000	40	750	865.89	Residual	14912.71		7	2130.39		
12	25,000	60	600	707.98	Lack of fit	5493.36		3	1831.12	0.7776	0.5646
13	25,000	40	750	806.53	Pure error	9419.36		4	2354.84		
14	25,000	40	750	918.98	Corrected total	3.687E^+05^		16			
1	250	40	40	822	Credibility analysis of the						
16	25,000	20	600	597.96	Standard deviation	Mean	CV (%)	*R^2^*	Adjust *R^2^*	Predicted *R*^2^	Adequacy precision
17	35,000	40	600	941.13	46.16	696.65	6.63	0.9596	0.9076	0.7217	13.32

Note: R2 = 0. 9596, R2adj = 0.9076, Pred R2 = 0.7217; *: significant (P < 0.05).

**Fig. 3:**
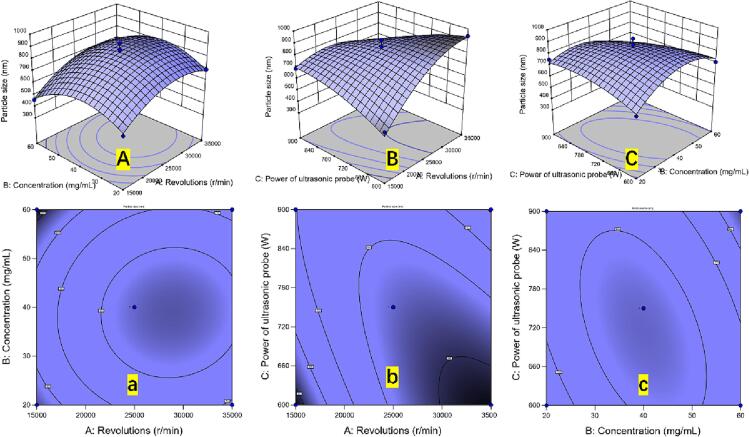
Response surface optimization of powder size prepared by various factors (A-C and a-c: interaction significant effect of different factors).

Design-Expert 10 software was used for the statistical analysis of the experimental data, and the following regression equation was obtained:

*Y = 843.96 + 91.17X_1_-19.51X_2_-25.99X_3_ + 11.42X_1_X_2_-144.56X_1_X3-80.32X_2_X_3_-118.07X_1_^2^-139.42X2-55.55X_3_2*.

where X_1_, X_2_, and X_3_ represent the homogenate rotation, ultrasonic power, and solution concentration, respectively, and Y denotes the particle diameter (nm). Positive coefficient factors favor a reduction in Y particle size, while negative coefficient values influence an increase in Y particle size. With Y (the size of the UPN) as the response value, the response surface and contour maps of the interaction between solution concentration (X_1_), homogenization speed (X_2_), and ultrasonic probe power (X_3_) were created (as shown in Fig. 3).

The influence of various factors on nobiletin particle size and their interaction can be reflected by the sharp change in the response surface and the shape and sparsity of the contours. The steeper the response surface is and the denser the contour is, the more significant the impact on the total output. As shown in the response surface diagrams in Fig. 3A and a, the liquid–solid ratio and microwave power curves are relatively steep, indicating that the particle size of the UPNs is strongly affected by the interaction between the two materials. A clear interaction between the two parameters and particle size is shown in Fig. 3A's 3-dimensional contour between concentration, revolution, and particle size, which is elliptical and dense. The particle size first increased as the rotation increased and then remained constant. The particle size initially increased as the concentration increased before decreasing. The contours of the power of the ultrasonic probe and revolution factors in Fig. 3B and b are steep, indicating obvious interactions between them. Fig. 3C and c show that the curve between the power of the ultrasonic probe and the concentration is an ellipse, indicating that their interaction has a significant influence on the UPN particle size. This ellipse indicates that there is a significant interaction between the two projection profiles.

For further analysis, the results obtained with Design-Expert 10.0.1 software showed that the optimal process for determining the particle size under the joint influence of ultrasonic power A, homogenization speed B, and solution concentration C was as follows: 670.39 W ultrasonic power, 16019.70 r/min homogenization speed, and 57.06 mg/mL solution concentration. Under these conditions, the minimum particle size of UPN was 505.55 nm. To verify the reliability of the process parameters, combined with the practical convenience of the operation, according to the predictions of the software and combined with the actual process, an ultrasonic power of 670 W, a homogenization speed of 16,000 r/min, and a concentration of 57 mg/mL were used for three repeated trials. The average particle size obtained was 521.02 nm, while the predicted value of the model was 505.55 nm, with an error less than 3 %. The response surface model verified the results well [5].

### 3.3 Microscopic characteristics

**Fig. 4:**
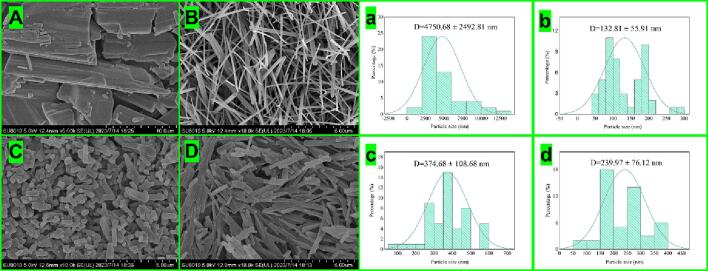
Scanning electron microscopy (SEM) images of the samples prepared by different methods (A: raw nobiletin, B: nobiletin ultrafine particles by solvent ultrasonication, C: nobiletin ultrafine particles from the solvent-homogenate sample and D: nobiletin ultrafine particles from the solvent-homogenate sample) and particle size distributions (a: raw nobiletin, b: nobiletin ultrafine particles from solvent ultrasonication, c: nobiletin ultrafine particles from the solvent-homogenate sample and d: nobiletin ultrafine particles from the solvent-homogenate sample).

The 4 samples were observed via SEM (Fig. 4). First, the original powder of nobiletin consisted of long strips or blocks (Fig. 4A, a), which indicated the occurrence of fracture. Compared to that of the other samples, the volume of RN is relatively large, approximately 4000–10000 nm.

Fig. 4B and b show the UPN prepared with the combination of an antisolvent and an ultrasonic probe. The sample is in a long and thin strip state, and some powders are similar to silk lines. Therefore, it is estimated that the vibration field during ultrasonication leads to narrow recrystallization of the sample, and a large amount of these powders are exposed, which greatly increases the specific surface area of the sample, resulting in a more uniform distribution of the powders and irregular distribution and overlapping of the particles. Fig. 4C shows the sample prepared with the combination of the antisolvent and ultrasonic homogenization methods. It is not difficult to see that the powder is uniform and ordered. Compared with those of the other preparation methods, the edges of the obtained sample are more regular, the particle size reaches 374.68 nm, and the sample has a better profile. Fig. 4D shows the sample prepared by the antisolvent/homogenization method. Although the particle size decreased to 239 nm (Fig. 4d), the powder exhibited severe accumulation and an irregular shape. Therefore, judging from the observed effect, we believe that the order of sample preparation should be as follows: Fig. 4C > Fig. 4B > Fig. 4D.

### 3.4 FTIR and XRD analysis

**Fig. 5:**
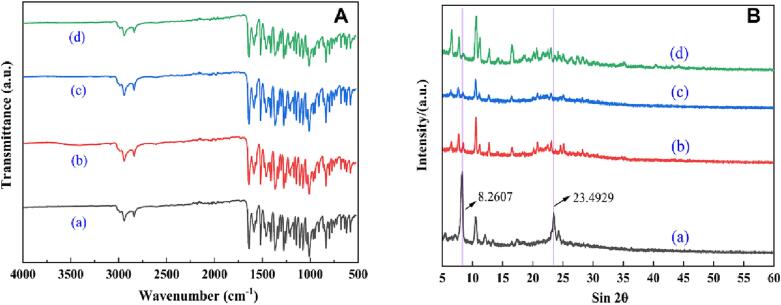
Properties of the powders (a: raw nobiletin, b: ultrafine particles of nobiletin determined by ultrasonication, c: ultrafine particles of nobiletin determined by the solvent-homogenate sample and d: ultrafine particles of nobiletin determined by the solvent-homogenate sample).

Fig. 5(a–d) shows the infrared spectral data of the sample. It is not difficult to see that for the tensile peak trend of RN (a) and the other samples (b-d), the amplitude of the peak is consistent, so it can be judged that all the samples exhibit the same trend; they all show a very consistent chemical structure. Therefore, we determined that different antisolvent methods did not change the original chemical structure of the sample, and the chemical properties of the sample did not change before or after preparation [23].

Fig. 5B shows the XRD analysis of the powder. First, the crystallization trends of the samples (4a-4d) are almost the same, but the peak height of the prepared sample decreases. At 8.26° and 23.49° of the sample, the peak height of the raw powder was the largest (Fig. 5B–(a)). However, the peak types of the UPN samples were not obvious at these two locations; we speculated that the RN was crystalline and that the treated UPN samples may have an amorphous state. This may be due to a decrease in peak strength caused by processing. G. Sodeifian et al. showed that a decrease in crystallinity is the cause of a decrease in the diffraction peak amplitude [24]. However, the peak strength of sample (c), which was prepared by the combination of antisolvent-ultrasound-homogenate, decreased the most, almost linearly. The crystalline state of the UPN samples basically disappeared, and the particle size decreased the most significantly.

### 3.5 Characterization of two samples by NMR

**Fig. 6:**
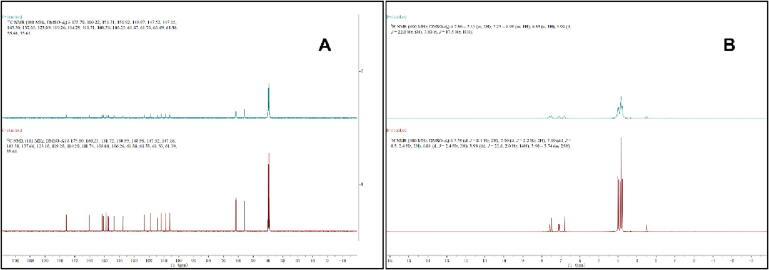
Characterization of two samples by NMR (A:Carbon spectrum, B:Hydrogen spectrum).

As is shown in the Fig. 6, the above 1H NMR and 13C NMR spectra, the ultrafine particles were very similar to those in the raw sample. The peak times and shapes of the raw nobiletin powder and ultrafine particles did not change; however, the peak heights were significantly different, indicating that the crystallinity of the ultrafine particles decreased, but the peak time did not change significantly. Therefore, it can be inferred that the structure of the polymethoxy flavonoid of the ultrafine particles did not change, but the crystal changed to a nonfixed state.

### 3.6 Detection of nobiletin samples by LC-MS

**Fig. 7:**
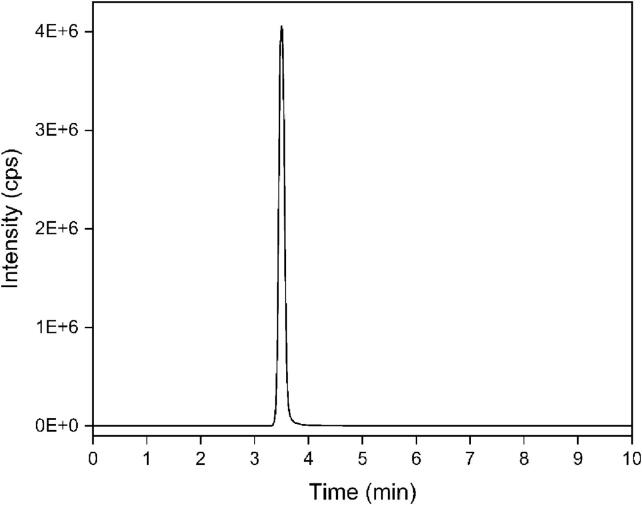
LC-MS detects the composition of the nobiletin.

After the generated powder sample was fully homogenized using ultrasonic treatment for 5 min, it was dispersed in deionized water. A 0.22 μm organic filter membrane was used for filtration. Then, LC-MS test was performed. The nobiletin we applied was a single compound with high purity (purity > 98 %). The test results of LC-MS showed that there was a standard spike at 3.5 min, and we could conclude that this ingredient was nobiletin (As is shown in the Fig. 7).

### 3.7 α-Glucosidase and porcine pancreatic lipase inhibitory activity

As shown in Table 3, α-glucosidase was inhibited in the RNs and UPNs, with acarbose serving as the positive control (the inhibition range was 28.21–77.95 %). The maximum inhibitory activity on α-glucosidase in UPN was 76.01 %, which was in the range of 15.63–500 μg/mL and was 18.43 % greater than the 57.58 % inhibitory activity in the RN sample. This difference may be because the dissolution rate of the micropowder per unit time was greater than that of the RN, and the inhibitory effects were greater.

**Table 3:** Inhibitory effects of nobiletin samples on α-glucosidase and porcine pancreatic lipase (PPL).

**Concentration (μg/mL)**	**Inhibitory activity of α- glucosidase (%)**			**Inhibitory activity of PPL (%)**		
	**RU**	**UPN**	**Acarbose**	**RN**	**UPN**	**Orlistat**
15.63	14.24	25.31	28.21	46.39	64.98	66.96
31.25	18.79	29.28	41.13	47.31	66.14	69.33
62.50	23.90	35.20	54.35	53.24	72.79	74.99
125	35.45	54.21	58.90	62.28	73.88	76.22
250	49.70	62.30	63.66	65.03	82.65	77.73
500	57.58	76.01	77.95	68.41	90.34	83.59
IC_50_ (μg/mL）	306.85	106.81	65.10	32.42	5.36	0.81

Note: The positive control for the inhibitory activity of α-glucosidase was acarbose, and the positive control for the inhibitory activity of PPL was orlistat. RN: raw nobiletin, UPN: ultrafine particles of nobiletin.

The range of solution concentrations in the PPL inhibition experiment was also 15.6–500 μg/mL (Table 3). The inhibitory activity of the RNs was 46.39–68.41 %, and the inhibitory efficiency of the micropowder was 64.98–90.34 %. This value was greater than that of α-glucosidase. In addition, at a maximum concentration of 500 μg/mL, the inhibition rate of the ultrafine powder on PPL was 21.93 % greater than that on RN [15]. Under the same conditions, the inhibitory effect of the ultrafine powder was reduced by 6.75 %. In summary, nobiletin has the potential to function as a natural enzyme inhibitor since, in the enzyme inhibition test, it functions similarly to plant essential oils, and its inhibitory capacity increases in a way that is positively related to concentration [25]. Similarly, the inhibitory effects of UPN on α-glucosidase and PPL were superior to those of both RN and the positive control, and the inhibitory effects were in the order of UPN > orlistat > RN.

## 4 Conclusion

The ultrafine powder for nobiletin used in this study was made using the ultrasonic-homogenate-aided antisolvent precipitation technique. Under ideal circumstances, the response index corresponds to the sample's smallest particle size. The optimal solution concentration and homogenization speed were 57 mg/mL and 16,000 r/min, respectively; the ultrasonic power was 670 W; and the antisolvent precipitation technique yielded an average minimum particle size of 521.02 nm. Comparing the sample made using this procedure to the raw sample did not alter its chemical composition, demonstrating that the crystallinity of the ultrafine powder decreased. To inhibit α-glucosidase and PPL, the UPN samples outperformed the RN samples in terms of the inhibition of PPL, which was inhibited by 500 mg/mL at 68.41 % for the original powder, 90.34 % for the ultrafine powder, and 83.59 % for the positive control, according to the data. The aforementioned studies serve as a reference for the practical use of nobiletin in plants and have practical implications for the creation of natural goods. In conclusion, the manufacture of superfine nobiletin powder using the ultrasound-homogenate-aided antisolvent approach can significantly increase the ability of the sample to block enzymes while also having a positive impact on future research.

## CRediT authorship contribution statement

**Xiaonan Zhang:** Writing – review & editing, Writing – original draft, Software, Resources, Project administration, Methodology, Funding acquisition, Formal analysis, Data curation, Conceptualization. **Yan Huang:** Software, Methodology, Formal analysis. **Siyi Huang:** Software, Methodology. **Wenyi Xie:** Software, Formal analysis. **Wenxuan Huang:** Software. **Yi Chen:** Methodology. **Qiyuan Li:** Formal analysis. **Fajian Zeng:** Formal analysis. **Xiongjun Liu:** Writing – review & editing, Writing – original draft, Formal analysis.

## Declaration of competing interest

The authors declare that they have no known competing financial interests or personal relationships that could have appeared to influence the work reported in this paper.
